# Analysis of the Bacterial Spectrum and Key Clinical Factors of Biliary Tract Infection in Patients with Malignant Obstructive Jaundice after PTCD

**DOI:** 10.1155/2022/1026254

**Published:** 2022-07-30

**Authors:** Dongjuan Xing, Weihua Song, Shaojuan Gong, Aimin Xu, Bo Zhai

**Affiliations:** Department of Interventional Oncology, Renji Hospital, School of Medicine, Shanghai Jiao Tong University, Shanghai, China

## Abstract

**Objective:**

To analyze the bacterial spectrum and key clinical factors associated with biliary tract infections following percutaneous transhepatic cholangial drainage (PTCD) for malignant obstructive jaundice (MOJ).

**Methods:**

This retrospective study comprised patients with MOJ who were treated with PTCD from 1^st^ June 2016 to 31^st^ December 2020. Patient clinical data, development of postprocedure biliary tract infections, spectrum of pathogenic bacteria, and drug sensitivity were analyzed, focusing on antibiotic drug resistance and identifying key associated risk factors for postoperative biliary tract infections.

**Results:**

Of the 528 study patients, 80 were diagnosed with postoperative biliary tract infections, 58 of whom had pathogenic bacteria detected in their bile samples. A total of 90 strains of pathogenic bacteria and 3 strains of fungi were detected; the top 4 were *Escherichia coli*, *Klebsiella pneumoniae*, *Enterococcus faecium*, and *Pseudomonas aeruginosa*. By univariate analysis, a positive bile culture following PTCD was closely correlated with both the location and degree of preoperative obstruction and the preoperative bilirubin level. Moreover, the results of logistic regression analysis concluded that complete obstruction and a high preoperative total bilirubin level prior to PTCD were independent risk factors for a positive bile culture following PTCD.

**Conclusion:**

Biliary tract infections following PTCD for MOJ were principally due to *Escherichia coli*, and bacteria in the bile were statistically more likely to be detected in patients with complete obstruction and high preoperative bilirubin levels.

## 1. Introduction

Malignant obstructive jaundice (MOJ) is caused by direct or indirect biliary obstruction typically from cancers of the liver, gallbladder, bile ducts, pancreas, and ampulla. These are primarily manifested clinically as jaundice, with evidence of biliary dilatation, significantly increased bilirubin, and yellow staining of body fluids. Malignant tumors that obstruct the biliary tract or the ampulla of Fatt can cause obstructive jaundice. These malignancies include cancer of the biliary tract, pancreatic head and neck cancer, cancer of the second part of the duodenum, and ampulla of Fatt. Biliary obstruction can alter normal physiology and affect multiple organ systems, including but not limited to cardiac, renal, blood, and liver dysfunction. Hyperbilirubinemia is a potential risk factor that may be associated with poor surgical outcomes. Percutaneous transhepatic cholangial drainage (PTCD) is an effective palliative treatment for markedly relieving jaundice and the associated symptoms of biliary obstruction [1–4], improving liver function, and extending patient survival. Nonetheless, it certainly qualifies as an invasive procedure, and the indwelling catheter may remain in place for weeks to months, which inevitably raises the risk of postoperative biliary tract infection. Such infections can be severe, sometimes leading to septic shock and even death [5]. The bacterial spectrum and key clinical factors of biliary tract infection following PTCD in MOJ have not been established. In the present study, we intended to determine the bacterial spectrum, antibiotic efficacy, and key clinical factors associated with biliary tract infections following PTCD for MOJ. This study contributes to the development of therapeutic strategies for malignant obstructive jaundice and the prevention and treatment of bacterial infection of the biliary tract.

## 2. Materials and Methods

### 2.1. Patients

This retrospective study comprised patients with MOJ treated with PTCD in our department from 1^st^ June 2016 to 31^st^ December 2020. The patient inclusion criteria were as follows: (1) diagnosed with MOJ by ultrasound, computed tomography (CT)/magnetic resonance (MR) imaging or pathology combined with symptoms and signs, and laboratory examinations; (2) successfully treated with PTCD; and (3) availability of complete clinical data. Patient exclusion criteria included the following: (1) severe cardiovascular or cerebrovascular diseases, hepatic or renal insufficiency, or coagulation dysfunction; (2) benign obstructive jaundice; (3) massive ascites; (4) diffuse biliary stricture; or (5) mental illness or cognitive impairment. The protocol of this study has been approved by the ethical committee of Renji Hospital, School of Medicine, Shanghai Jiao Tong University.

### 2.2. PTCD Procedure

PTCD was successfully conducted in all patients. Under the guidance of B-mode ultrasound, the puncture needle was inserted through the liver into the dilated biliary duct under digital subtraction angiography (DSA). The guide wires were exchanged, a 6F to 7F sheath was introduced, and cholangiography was performed to define the degree and location of the stenosis. Next, a 5F catheter was introduced to probe the stenosis and determine the most appropriate therapeutic intervention. The decision was primarily based on whether the stenosis could be transgressed and if surgical intervention was feasible. If the stenosis could be transgressed, (1) internal-external drainage catheters would be inserted into the bile duct if surgery was feasible or (2) a biliary stent and an external drainage catheter would be implanted if surgery was contraindicated. Patency of the biliary stents for internal drainage was assured, and the external drainage catheter was clamped 3 days after the procedure. If the stenosis could not be transgressed, an external drainage catheter would be inserted.

### 2.3. Diagnosis of Biliary Tract Infections after PTCD

As specified in the *Guidelines for Diagnosis and Treatment of Acute Biliary Tract Infections (2021)*, the diagnostic criteria for biliary tract infections include the following: (A) systemic inflammation—(1) fever (body temperature > 38°C) and/or chills and (2) laboratory examination: white blood cell count > 10 × 10^9^/L and C‐reactive protein ≥ 1 g/L; (B) cholestasis—(1) jaundice (total bilirubin ≥ 34.2 *μ*mol/L) and (2) alkaline phosphatase (U/L) > 1.5 × upper limit of normal, *γ*‐glutamyltranspeptidase (U/L) > 1.5 × upper limit of normal, AST (U/L) > 1.5 × upper limit of normal, and ALT (U/L) > 1.5 × upper limit of normal; and (C) imaging examination—(1) biliary duct dilatation and (2) pathogenic factors detected by imaging (stenosis, stones, tumors, stents, etc.) [6].

### 2.4. Retention of Bile

Bile was sampled either prior to drainage catheter removal or when biliary tract infection was suspected. By criteria, biliary tract infections would be suspected if patients had 1 criterion in A and +1 criterion in B or C. Bile samples were obtained according to strict sterile procedures as follows: (1) informed consent was obtained; (2) sterile equipment was prepared; (3) personnel followed the 6-step handwashing method; (4) sterile drapes were spread, connections of drainage catheters were strictly sterilized, and a sterile syringe was connected to drainage catheters to extract 5-10 mL of bile; and (5) the bile was injected into sterile tubes and sent for gram stain, cultures for bacteria+fungi, and drug sensitivity tests.

In this study, data collected included the following: (1) basic patient data including age and gender; (2) preprocedural indications, including the site and degree of obstruction, duration of preoperative jaundice, and preoperative bilirubin level; (3) procedure-associated variables, including mode of drainage and volume and number of PTCD catheters; and (4) bacterial culture and drug sensitivity test results of infected cases.

## 3. Statistical Analysis

SPSS 22.0 (IBM SPSS Statistics, USA) was used for data processing and statistical analysis. Count data were expressed by frequency and percentage. The *χ*^2^ test or Fisher's exact test was conducted for comparison between groups. Binary logistic regression analysis was performed to assess the risk factors for positive bile cultures. The two-sided *P* less than 0.05 was set as significant difference.

## 4. Results

### 4.1. Biliary Tract Infections after Interventional Therapy

This study included 528 patients with MOJ treated with PTCD in our department from 1st June 2016 to 31st December 2020. There were 334 males and 194 females, with an age range of 20-96 years (mean, 65.5 ± 11.73). Of the 528 study patients, 80 were diagnosed with postoperative biliary tract infections, with an infection rate of 15.2%. Bacteria were detected in 58 bile samples (detection rate, 72.5%; Table 1) including 93 pathogens: 51 gram-negative and 39 gram-positive bacteria and 3 strains of fungi.

### 4.2. Results of Drug Sensitivity of the Main Pathogenic Bacteria

Dominant among the 90 strains of pathogenic bacteria were *Escherichia coli*, *Klebsiella pneumoniae*, *Enterococcus faecium*, *Pseudomonas aeruginosa*, and *Enterococcus faecalis*. The drug sensitivity results of the three principal gram-negative bacteria (*Escherichia coli*, *Klebsiella pneumoniae*, and *Pseudomonas aeruginosa*) and two gram-positive bacteria (*Enterococcus faecium* and *Enterococcus faecalis*) were statistically analyzed (Tables 2 and 3).

The sensitivity rate of *Escherichia coli* to tigecycline, imipenem, meropenem, piperacillin tazobactam, cefoperazone sulbactam, and amikacin exceeded 90%; that of *Klebsiella pneumoniae* to amikacin, tigecycline, imipenem, meropenem, piperacillin sulbactam, and cefoperazone sulbactam was 75-90%; and that of *Pseudomonas aeruginosa* to amikacin, cefoperazone sulbactam, cefepime, gentamicin, tobramycin, and piperacillin sulbactam was 100%. *Escherichia coli*, *Klebsiella pneumoniae*, and *Pseudomonas aeruginosa* exhibited high resistance rates to ampicillin, ceftriaxone, and cefazolin. Moreover, gram-positive bacteria, *Enterococcus faecium* and *Enterococcus faecalis*, showed 100% sensitivity to linezolid, tigecycline, teicoplanin, and vancomycin.

### 4.3. Analysis of Associated Risk Factors for Positive Biliary Cultures in MOJ Patients Treated with PTCD

There were no statistical differences (*P* > 0.05) comparing the positive versus the negative bile culture groups with regard to gender, age, drainage mode, number of PTCD catheters, drainage volume, days of preoperative jaundice, or history of diabetes. In contrast, the site and degree of biliary obstruction and preoperative bilirubin level were dramatically and statistically lower in the negative bile culture group (*P* < 0.05, Table 4).

With gender, age, drainage mode, degree of obstruction, site of obstruction, number of PTCD catheters, drainage volume, days of preoperative jaundice, preoperative total bilirubin level, and history of diabetes as covariates and whether bile cultures were positive as a dependent variable, binary logistic regression analysis (forward: Wald) was conducted. The degree of obstruction and the preoperative bilirubin level were demonstrated to be independent risk factors for positive bile cultures in MOJ patients treated with PTCD (Table 5).

## 5. Discussion

Biliary tract infection, a common and serious complication of MOJ, is a critical factor that must be borne in mind when considering the risk versus benefit of proceeding with PTCD [3, 7–10]. At present, antibiotics are routinely used to prevent infections following interventional decompression of MOJ. However, the abuse of antibiotics has gradually caused evolution of drug resistance to pathogenic bacteria. Hence, to highlight both the spectrum of pathogenic bacteria and antibiotic drug resistance in patients with MOJ complicated with infections following interventional therapy is vitally important, to appropriately use and select antibiotics and reduce mortality.

Our data of 93 strains of pathogens, including 55% gram-negative (predominantly *Escherichia coli*) and 42% gram-positive bacteria (predominantly *Enterococcus faecium*) and 3% fungi, are quite consistent with previous literature reports [11, 12]. Therefore, bacteria that cause biliary tract infections following interventional therapy for malignant obstruction are mostly derived from the intestinal tract. Logically, the bacterial course retrogrades along PTCD catheters and stents, through the duodenal papilla and into the biliary system. In the present study, the bacterial culture and drug sensitivity results revealed that gram-negative bacteria exhibited high resistance rates to penicillin, most cephalosporins, and quinolones, but low resistance rates to *β*-lactamase inhibitor compound preparations, carbapenems, and aminoglycosides. Additionally, the resistance rates of gram-positive bacteria to penicillin, clindamycin, erythromycin, and quinolones were higher, whereas resistance to linezolid, tigecycline, teicoplanin, and vancomycin was lower. Moreover, statistical analysis demonstrated that a large proportion of bile cultures had multi-drug-resistant bacteria: 11 strains of extended-spectrum *β*-lactamase- (ESBL-) producing gram-negative bacteria, 8 strains of carbapenem-resistant gram-negative bacteria, 8 strains of methicillin-resistant coagulase-negative staphylococci, 2 strains of methicillin-resistant staphylococci, and 1 strain of vancomycin-resistant enterococcus. In fact, in some patients, 2 or 3 kinds of pathogenic bacteria were cultured in the bile. Patients with MOJ often have predisposing risk factors that complicate their management, such as weakened immunity [13], long hospital stay, and frequent use of antibiotics. Therefore, the use of antibiotics should be carefully considered, most reliably prescribed on the basis of bacterial culture and drug sensitivity tests in combination with clinical experience [14, 15].

As denoted by the results of this study, the site and degree of preoperative biliary obstruction and the preoperative bilirubin level were closely correlated with the rate of positive bile cultures following interventional therapy. Furthermore, complete obstruction and high preoperative total bilirubin levels were independent risk factors for the rate of positive bile cultures following interventional therapy. In this study, biliary obstruction was classified into three types: hilar, midcommon bile duct, and lower common bile duct. The higher the level of obstruction, the higher the probability of postoperative biliary tract infections. Because obstructing cancers in the hilum can obstruct multiple intrahepatic bile ducts, it can be difficult to achieve adequate drainage of all obstructed ducts with puncture and drainage. Implantation of multiple stents might further aggravate stenosis of smaller branches with consequent higher risk of infection. Bile is an ideal culture medium, and complete obstruction increases the incidence of cholangitis prior to operation. Importantly, if interventional drainage is incomplete, it can disseminate bacteria in the biliary tract, triggering biliary tract infections. Total bilirubin is a crucial liver function index. The poorer the liver function, the lower the external stimulus-resistance capacity and the higher the incidence of infections [16]. Currently, risk factors for biliary tract infections following interventional therapy for MOJ have been rarely reported, and the conclusions vary [17, 18]. This appears to relate mainly to limitations of both data and retrospective studies. Additional large cohort studies or prospective studies would be optimal.

To conclude, bacteria responsible for biliary tract infections in patients with MOJ following radiologic decompressive interventions are mostly derived from the intestinal tract, dominated by gram-negative bacteria. In addition, the use of antibiotics should be carefully considered and selected based on bile cultures and drug sensitivities. This is of particular importance in patients with documented high-risk factors including upper biliary obstruction, complete obstruction, and preoperative high bilirubin levels.

## Figures and Tables

**Table 1:** Distribution of pathogenic bacteria in bile samples of patients.

Species of bacteria	Number of strains (*n*)	Constituent ratio	Species of bacteria	Number of strains (*n*)	Constituent ratio
Gram-negative bacteria	51	54.84%	Gram-positive bacteria	39	41.94%
*Escherichia coli*	15 (8 ESBLs)	16.12%	*Enterococcus faecium*	11	11.83%
*Klebsiella pneumoniae*	12 (3 ESBLs)	12.90%	*Enterococcus faecalis*	5	5.38%
*Pseudomonas aeruginosa*	6	6.45%	*Staphylococcus epidermidis*	5	5.38%
*Stenotrophomonas maltophilia*	5	5.38%	*Staphylococcus haemolyticus*	4	4.30%
*Enterobacter aerogenes*	2	2.15%	*Enterococcus gallinarum*	3 (VRE 1)	3.23%
*Xanthomonas fusca*	1	1.08%	*Streptococcus angina*	3	3.23%
Pseudomonad	1	1.08%	*Staphylococcus aureus*	2 (MASA 2)	2.15%
*Proteus mirabilis*	1	1.08%	*Streptococcus sanguis*	2	2.15%
*Ochrobactrum anthropi*	1	1.08%	*Enterococcus durans*	1	1.08%
*Enterobacter cloacae*	1	1.08%	*Enterococcus hirae*	1	1.08%
*Bacillus mucilaginosus*	1	1.08%	*Staphylococcus cohnii*	1	1.08%
*Citrobacter brucei*	1	1.08%	*Enterococcus raffinose*	1	1.08%
*Klebsiella oxytoca*	1	1.08%	Fungus	3	3.23%
*Citrobacter freundii*	1	1.08%	*Candida glabrata*	2	2.15%
*Acinetobacter ursingii*	1	1.08%	*Candida krusei*	1	1.08%

**Table 2:** Drug resistance of the main gram-negative bacteria.

Antibiotic	*Escherichia coli* (15 strains)		*Klebsiella pneumoniae* (12 strains)		*Pseudomonas aeruginosa* (6 strains)	
	Drug-resistant strain (*n*)	Drug resistance rate	Drug-resistant strain (*n*)	Drug resistance rate	Drug-resistant strain (*n*)	Drug resistance rate
Amikacin	1	6.67%	1	8.33%	0	0.00%
Aztreonam	6	40.00%	6	50.00%	1	16.67%
Ciprofloxacin	8	53.33%	7	58.33%	1	16.67%
Cefoperazone sulbactam	1	6.67%	3	25.00%	0	0.00%
Cefuroxime	8	53.33%	6	50.00%	—	—
Cefepime	3	20.00%	5	41.67%	0	0.00%
Gentamicin	6	40.00%	4	33.33%	0	0.00%
Levofloxacin	7	46.67%	7	58.33%	1	16.67%
Paediatric compound sulfamethoxazole tablet	8	53.33%	4	33.33%	5	83.33%
Tobramycin	4	26.67%	3	25.00%	0	0.00%
Ampicillin	14	93.33%	12	100.00%	5	83.33%
Ceftazidime	5	33.33%	6	50.00%	2	33.33%
Ceftriaxone	8	53.33%	6	50.00%	5	83.33%
Cefotetan	1	6.67%	3	25.00%	4	66.67%
Cefazolin	9	60.00%	6	50.00%	6	100.00%
Imipenem	1	6.67%	3	25.00%	4	66.67%
Meropenem	1	6.67%	3	25.00%	2	33.33%
Ampicillin sulbactam	10	66.67%	6	50.00%	5	83.33%
Tigecycline	0	0.00%	2	16.67%	—	—
Piperacillin tazobactam	1	6.67%	3	25.00%	0	0.00%

**Table 3:** Drug resistance of the main gram-positive bacteria.

Antibiotic	*Enterococcus faecium* (11 strains)		*Enterococcus faecalis* (5 strains)	
	Drug-resistant strain (*n*)	Drug resistance rate	Drug-resistant strain (*n*)	Drug resistance rate
Ampicillin	8	72.73%	0	0.00%
Erythromycin	9	81.82%	2	40.00%
Linezolid	0	0.00%	0	0.00%
Moxifloxacin	10	90.91%	2	40.00%
Penicillin G	8	72.73%	2	40.00%
High-concentration streptomycin	4	36.36%	1	20.00%
Tigecycline	0	0.00%	0	0.00%
Clindamycin	10	90.91%	5	100.00%
High-concentration gentamicin	3	27.27%	1	20.00%
Levofloxacin	10	90.91%	2	40.00%
Minocycline	1	9.09%	0	0.00%
Quinupristin/dalfopristin	3	27.27%	5	100.00%
Teicoplanin	0	0.00%	0	0.00%
Vancomycin	0	0.00%	0	0.00%

**Table 4:** Univariate analysis of indicators related to positive bile cultures.

	Related indicator	Case (*n*)	Number of positive strains	Positive rate	*χ* ^2^	*P*
Gender	Male	59	44	74.58	0.49	0.649
	Female	21	14	66.67		
Age (years old)	≤60	23	16	69.57	0.14	0.879
	>60	57	42	73.68		
Degree of obstruction	Incomplete obstruction	41	16	39.02	21.73	0.013
	Complete obstruction	39	33	84.62		
Site of obstruction	Hilar part	36	31	86.11	6.99	0.041
	Middle part of the common bile duct	8	6	75.00		
	Lower part of the common bile duct	36	21	58.33		
Drainage mode	Intermittent drainage	9	9	100.00	4.94	0.876
	External drainage	49	32	65.31		
	External drainage+stent	22	17	77.27		
PTCD catheter	1	61	41	67.21	3.91	0.618
	2	14	13	92.86		
	3	5	4	80.00		
Drainage volume (mL)	≤300	21	13	61.90	1.60	0.532
	>300	59	45	76.27		
Days of preoperative jaundice (d)	≤20	54	38	70.37	0.38	0.987
	>20	26	20	76.92		
History of diabetes	Yes	21	14	66.67	0.57	0.065
	No	59	7	11.86		
Preoperative total bilirubin level (*μ*mol/L)	≤200	43	27	62.79	0.05	0.013
	>200	37	31	83.78		

**Table 5:** Logistic regression analysis (enter method of regression) of positive bile cultures.

Indicator	*β*	SE	Wald	*P*	OR	95% CI
Degree of obstruction	-1.316	0.565	5.418	0.020	0.268	0.089-0.812
Preoperative total bilirubin	0.004	0.002	3.971	0.046	1.004	1.000-1.008

## Data Availability

We declare that all data were provided in this manuscript.
